# Hospital and Institutional News

**Published:** 1912-03-16

**Authors:** 


### HOSPITAL AND INSTITUTIONAL NEWS.

### APPLICATIONS FOR GRANTS FROM THE KING'S FUND.

We are asked to state that hospitals in the county of London, or within nine miles of Charing Cross, desiring to participate in the grants made by King Edward's Hospital Fund for the year 1912 must make application before March 30 to the hon. secretaries, 7 Walbrook, E.C. Applications will also be considered from convalescent homes which are situated within the above boundaries, or which, being situated outside, take a large proportion of patients from London. Applications will also be considered from sanatoria for consumption which take patients from London, or which are prepared to place beds at the disposal of the Fund for the use of patients from London hospitals.

### SIR FREDERICK TREVES' RETIREMENT.

The news that Sir Frederick Treves has determined to retire from active public work on the Army Medical Advisory Board has come as a surprise to the public. But it should be remembered that Sir Frederick has been connected with this work for eleven years, since indeed it was first undertaken. His term of office was continual, and his activities extended to a personal inspection of practically all the military hospitals throughout the Empire. Since his appointment many important improvements have been effected in the Army Medical Service: the military hospitals have been centralised, a system enabling soldiers with slight injuries to be treated in barracks has been introduced, and the Millbank Royal Army Medical College has been founded. Sir Frederick's institutional interests, however, will be maintained by his connection with King Edward's Sanatorium at Midhurst, the Officers' Convalescent Home at Osborne, and the London Radium Institute.

### LONDON'S DEPUTY MEDICAL OFFICER.

Mr. William Butler has been appointed by the London County Council, at its meeting on March 5, deputy medical officer of health and deputy school medical officer for the County of London at a salary of £1,000 a year. There were thirty candidates for the post, ten of whom were selected to be interviewed by the Establishment Committee. Mr. Butler, who is the medical officer of health and school medical officer to the Urban District Council of Willesden, is an M.B. of Glasgow, and was formerly medical superintendent of the Willesden Isolation Hospital. Mr. Butler has contributed several articles on public health subjects to the technical papers.

### HOSPITALS AND THE COAL STRIKE.

The position of hospitals at the moment is much the same as that of every other large institution; at present on the average the hospitals throughout the country appear to have about a month's coal supply, and any serious anxiety cannot be generally reported at present. It may be worth while placing this on record, for the lay papers have been doing their best, for party purposes, to exaggerate a premature alarm. On the other hand, however, it cannot be stated too strongly that now, before it is too late, is the time for subscribers to rally to the support of the hospitals, and to increase their subscriptions, so that should the coal dispute be seriously prolonged they may be in a better position to meet their difficulties. This is the point to be emphasised, and we hope that every hospital secretary is pressing on his subscribers, and on the public, the urgent need of forestalling a threatened coal-famine by subscribing liberally while there still remains coal to be had.

### LONDON'S NEW MENTAL HOSPITAL.

The Mental Hospitals Committee of the London County Council is about to spend £12,400 in preliminary work on the site for the eleventh London County Mental Hospital on the Horton estate. The question of the number of blocks which should be provided in the first instance is left over for future consideration, and the works now proposed are, from their nature, general to the complete mental hospital and such as cannot be carried out in sections. They include provision of fencing, piping a watercourse, the provision of main sewer, and the provision of an 18-foot road from Horton Lane to the main entrance.

### THE AIM OF COTTAGE HOSPITAL MORTUARIES.

In opening a bazaar in aid of the funds of Tonbridge Cottage Hospital the other day, Sir Henry Burdett laid particular stress upon the aim and standard which cottage hospitals ought to uphold in their mortuaries. "You want £500," he said, "because you have a debt of £120, and because you need certain repairs, and because you must have a mortuary. At the present time you have an apology for a mortuary. It is not a mortuary at all. Now I attach great importance to the mortuaries of cottage hospitals. For a cottage hospital should set an example in hygiene to the whole community which it serves, and it should set also an example of reverence for the dead. It is extraordinary to me what an absence of this reverence one sometimes meets with in public institutions. Here you have a delightful hospital, and I venture to say, if you could apply such a word to this branch of hospital work, you ought to have a delightful mortuary. I hope, therefore, that the good people of Tonbridge will not be content to have any apology for a mortuary—the sort of coal-hole with a top that is sometimes found—but that you will have a proper mortuary with a mortuary chapel, where friends may see their dear departed. You should also have an additional room for medical purposes."

### WHERE COTTAGE HOSPITALS MUST SET AN EXAMPLE.

These are strong words and set a high standard. If some say too high a standard in days when subscriptions are hard to secure, the answer is that it is the same standard merely which is universally insisted upon in every other department of modern hospital work. We have little doubt that the Hospital's president, Mr. O. E. d'Avigdor Goldsmid, J.P., to whose generosity and personal service Sir Henry Burdett alluded in another part of his speech, will have welcomed this expert pleading for a cause of expenditure that is not nearly as much respected as it ought to be. The honorary secretary, Mr. J. W. Little, whose work in connection with the architecture of the institution as well as with its administration has shown great ability, will feel also that that part of the appeal which needed all the authoritative backing it could get, has been very wisely emphasised. It is because similar emphasis ought to be placed upon the mortuary everywhere in order that a general standard, especially in cottage hospitals, may be procured, that we have devoted so much attention to this part of the proceedings. The moral is one which every cottage hospital ought to learn.

### AN UNFORTUNATE INCIDENT AT SHEFFIELD.

The vast majority of complaints and criticisms levelled at the treatment afforded in the voluntary hospitals turn out on expert investigation to be baseless. Usually such criticisms are based upon imperfect information; sometimes upon rank ingratitude or downright perversion of the truth. Appreciating this to the full, it is also to be granted that cases do occur in which individual hospital officers fail to follow the best possible course. When such an event happens, it is perhaps natural that excuses should be offered. Without fuller information than that given by brief reports of an inquest held lately in Sheffield, it is difficult to assess exactly what blame, if any, attaches to the Sheffield Royal Hospital for the unfortunate death of a man who was struck in the chest by a flying mass from a burst grindstone. The patient, a man of fifty-five, was bandaged up at the hospital, and sent home in a cab: he died at home two days later, having seven broken ribs. Any experienced resident medical officer would, beyond doubt, have detained this man; or, if he had no vacant available bed, would have sent him on at once to some other institution. The secretary of the Royal Hospital, in a communication to the Press, endeavours to explain away this omission by stating that it is very unusual to detain sufferers from broken ribs; and that the sitting posture is preferable to recumbency during their removal. Quite true; but then seven ribs is a very unusual number to break, and, in fact, constitutes a very serious accident in the prime of life, let alone at fifty-five. With some regret we feel obliged to characterise the secretary's statement as unsatisfactory: a frank admission of a serious error in judgment would be better than this. Finally, it is worth noting the secretary also announces authoritatively that "last night there were three patients in the surgical wards more than we had beds for"; and this again is an episode on which we cannot congratulate either him or his board.

### WHAT A LADIES' GUILD CAN DO.

A capital speech was delivered the other day at the Nelson Hospital, Wimbledon, the buildings of which are nearly finished, by Miss Catharine E. A. Thorpe, on the functions of ladies' guilds. Miss Thorpe's first point was how much a hospital gained from a ladies' guild if the guild had such auxiliaries as linen, flower, and poultry leagues and a mending-party. At a mending-party, ladies seeing the condition of articles given them to repair often were moved to give new ones. Flowers made immense difference in a ward, and yet the ordinary hospital funds could not be devoted to their purchase; and presents of game and poultry not only saved the hospitals' bills, but made a welcome change of diet for the patients. Finally, a ladies' association could provide comforts for the staff which would otherwise be lacking. These are the arguments in a nutshell, and were listened to with great interest not only by the seventy odd members of the guild but many gentlemen also, including Mr. Thomas Raffles Hughes, K.C., who presided; Colonel Thomas Mitchell, C.B., president of the hospital; Alderman Summerhays, chairman; Mr. D. D. Kirkaldy, honorary secretary, and the Rev. D. C. Macgregor. These gentlemen as hospital workers no doubt had realised the fact that the value and work of a ladies' guild is never better shown than when a lady who is also a fluent speaker can be found to expound it.

### A NEW SCHOOL OF TROPICAL MEDICINE.

The Liverpool School of Tropical Medicine is still in its early 'teens, and the London School is even younger still, we believe. Yet both these institutions have already acquired reputations which are world-wide, and both attract students not only from Great Britain and the Continent, but also from our own Colonies and from those of other nations. It is therefore interesting to learn, on the authority of the *Indian Medical Gazette*, that a school of tropical medicine is soon to be established in Calcutta, and the project is so far advanced that the necessary buildings have already been taken in hand. The advantage of stationing a school of tropical medicine actually in the tropics, and of having an available supply of clinical material which may justly be called unlimited, is so great that with ordinary good management the success of the school must be rapid and apparent. It may be noted further, as evidence of the interest which the Government of India is taking in tropical medicine, that a malaria bureau was last year inaugurated at Amritsar, and that an official journal named *Paludism* is also being published.

### A GREAT TEACHER OF ANATOMY.

Dr. Alfred Harry Young, Emeritus Professor of Anatomy at the Victoria University of Manchester, has died, after a long illness, in his sixtieth year. At Edinburgh his reputation was so high that he was induced to accept a post as demonstrator and assistant lecturer in anatomy at Owens College, Manchester, under Professor Morrison Watson.

This was in 1877, and two years later Dr. Young became pathological registrar at the Royal Infirmary; after filling the posts of surgical and medical registrar there, he was elected honorary surgeon to the Manchester Hospital for Consumption and Diseases of the Chest and also to the Salford Royal Hospital. After being disappointed in securing election on the staff of the Royal Infirmary, he was in 1885 appointed to the Chair of Pathology at Owens College, and soon afterwards became the Dean of the School. As a teacher of anatomy he strove hard to simplify the enormous difficulties of the subject, and his diagrams were marvels of lucidity. On comparative anatomy he conducted many important researches, which were conducted at a menagerie near Manchester, and in the field of embryology he was recognised as one of the foremost teachers. It may be recalled that Dr. Young strongly opposed the admission of women students to the School of Medicine, but his attitude to the female students personally was always characterised by great kindness and an entire absence of prejudice.

### THE HOME SECRETARY AND A MENTAL HOSPITAL.

Mr. A. O. Goodrich, Chairman of the Mental Hospitals Committee of the London County Council, has announced that the Home Secretary has ordered an inquiry into the case of William Ball, the suffragist, who was admitted to Colney Hatch Mental Hospital on February 12 and was discharged on the following day. Mr. A. O. Goodrich stated that Ball was discharged at the instance of his wife, in order that he might become a private patient. A wife was empowered by the Lunacy Act of 1890 to take their course.

### THE REMOVAL OF PATIENTS FROM ISOLATION HOSPITALS.

An interesting point was raised at a recent meeting of the Chorley Town Council when Councillor Sandham moved that in the opinion of the Council the Chorley Joint Hospital Board ought to convey to their homes patients on their discharge from the institution. He urged that patients when leaving often could not afford a conveyance, and that by a provisional order the Joint Hospital Board had power to convey patients home. The Chairman of the Joint Hospital Board denied the application of the provisional order to this purpose, and urged the expense of the proposal. To this Councillor Sandham retorted that as patients were compelled to be taken to the isolation hospital they should be taken home. A common-sense view of the matter, whatever the terms of the provisional order may be.

### A COT WITH A WONDERFUL HISTORY.

Cots with a history would form an admirable subject for a historical monograph, and throw many interesting sidelights on the development of the voluntary system. A recent instance worth recording is that of the cot given by Mr. and Mrs. Edward Belfield to the Ashton District Infirmary. The governors of this institution were pleasantly surprised to receive a promise from Mr. and Mrs. Belfield to give £1,000 to endow a cot in the Ashton Infirmary. The surprise felt by the governors was partly due to the fact that the generous donors were known to have been employed in the local cotton-factory for the greater part of their lives, and consequently were not expected to be so well off as this gift, we are glad to think, shows them to be. Mr. Belfield, who is in his seventy-eighth year, has spent his life as what is technically called a "twister," while Mrs. Belfield is understood to have worked as a weaver. According to the local paper, which has published an interview with Mr. Belfield, the first year of their married life their earnings amounted to £67 19s., of which nearly £35 was "put away," and their annual rate of expenditure does not appear to have increased for the next twenty years. Mrs. Belfield retired in 1880, and Mr. Belfield in 1884, since when at any rate they have enjoyed, we understand, holidays in Scotland and elsewhere stretching on occasion to seven weeks at a time. It had been their intention to endow the cot on the occasion of their golden wedding, which will take place in two years' time, but, feeling that the halest life is mortal, Mr. Belfield resolved not to delay his pet project any longer, especially as this year is the Jubilee year of the Ashton Infirmary. The cot will be known not as the Belfield cot, but as the Jubilee cot, and its gift is alone enough to make the Jubilee year memorable. We have no hesitation in saying that one of the most honoured cots with a history which the voluntary system can show will be that given by Mr. and Mrs. Belfield.

### BIRMINGHAM'S FINE ACHIEVEMENT.

Sir Hallewell Rogers has been congratulating the members of the Birmingham Hospital Saturday Fund upon the fine total of almost £22,000 which has been raised during the past twelve months. It is fourteen years, he said recently, as chairman of the Fund, since the annual increase, which was some £1,500, had been so large. Comparing Birmingham's total with that of other Saturday Funds, he remarked "Birmingham beats Liverpool and Manchester combined by no less than £6,000." That is undoubtedly a fine achievement, and every credit must be given to the members and organisers of the Fund, among whom Mr. W. S. Aston, the honorary secretary, deserves special mention. We may add that Mr. Aston has been made an alderman, from which the Fund as well as the Council should again benefit.

### NEW PRESIDENT OF FALMOUTH HOSPITAL.

Mr. A. Newland Deakin, the headmaster of Falmouth Grammar School, has been appointed president of the Falmouth Hospital in succession to Major Luard. We understand that it is seven years since Major Luard became associated with hospital work, and he has held the office of president for two of them, during which time he has been a generous donor as well as a keenly interested worker for the hospital. If the completion of the new theatre, which was the local memorial to King Edward, had not necessitated the closing of the hospital for one month, the year's deficit of £57 would have been much greater, though the Friendly Societies' contributions had not come in when the report was presented. This means that Mr. Newland Deakin takes up the office of president at a not unduly anxious time. The King Edward memorial has been carried through, and the disorganisation caused by a change of matrons is now a thing of the past. If the public will only take Major Luard's appeal to heart, the secretary, Mr. Noel Lake, should be able to report satisfactory progress at the end of the ensuing twelve months.

### AN ALMONER FOR IN-PATIENTS.

The appeal of Miss Julia Chaplin and Miss Paulina Pepys-Cockerell in support of a scheme for the after-care of in-patients leaving the Great Ormond Street Hospital for Sick Children, virtually creates a new post in hospital work. These two ladies for the past three years have been reporting every child leaving the wards to whatever agency in its home district is best able to provide any after-care required, whether to the district nurse or to the Invalid Children's Aid Association, as the case may be. They have in each case reported the nature of the child's illness, received in turn reports of its progress, and done their best to supervise all cases the needs of which found no agencies to provide for them. These duties Miss Chaplin and Miss Pepys-Cockerell wish to hand over to a special worker, who has become a necessity to the hospital now that the pioneer voluntary workers have been compelled to give up this work. The board of management, indeed, officially endorses the scheme, for which £5,000 is needed as a trust fund to provide the necessary salary and expenses of the worker. The new official, who might be described perhaps as an almoner for in-patients, will of course require a special training in economic and social work. The hospital's treasurer, Mr. J. F. W. Deacon, will be one of the joint treasurers of the fund, for which (we are glad to say) a moiety has been already subscribed. This is only the latest step in making the hospital a centre of many health and reconstructive agencies, and should supply the need "for a link between the convalescent home and existing organisations" which Sir William Osler pleads for in supporting the proposal.

### A HOSPITAL PRESIDENT'S RECORD.

Sir Ernest Tritton, Bart., President of the Norwood Cottage Hospital, confessed to a fine record at the recent annual meeting. He said he had inherited his father's interest in the institution, which the latter had helped to found, and that he himself had been present at every meeting since 1892. That is a fact to be proud of, and the personal service to which it bears witness is no doubt one of the important causes of the successful annual meeting the other day, which Sir Ernest Tritton described as one of the best they had ever had. No doubt, too, the keen interest of the working-men to which he alluded is another proof of the personal service which lies at the basis of all hospital success. Mr. W. W. Garnham is again honorary secretary, Mr. W. H. Hills treasurer, and Mr. G. H. Finch is among the new vice-presidents for the year. More than one speaker referred to the work of the Rev. W. B. Taylor, who has been re-elected to the board of management. This annual meeting had real value as illustrating the vital importance of the personal element in hospital work.

### IMPORTANT BIRMINGHAM APPOINTMENTS.

Dr. Robert Cunyngham Brown, at present medical officer of his Majesty's prison, Birmingham, has been appointed by the Secretary for Scotland Deputy Commissioner in Lunacy for Scotland in the room of the late Dr. J. F. Sutherland. Dr. Brown was formerly medical officer at the Parkhurst Prison, Isle of Wight. Another appointment of interest to the hospital world of Birmingham is that of Professor Peter Thompson, who has been appointed Dean of the Medical Faculty of Birmingham University. Professor Thompson occupies the chair of anatomy at Birmingham, and succeeds Professor Barling, who has held the post of Dean of the Medical Faculty for seven years.

### NEW HOSPITAL SECRETARY AT TORQUAY.

Mr. E. J. V. Husey has been appointed honorary secretary to the Torbay Hospital, Torquay. Mr. Husey, who has already shown his interest in the hospital in many ways, succeeds Mr. C. S. Wollen, who held that post for ten years, and now continues his services as a member of the board of management. The secretary is still Mr. H. J. Packe. Mr. Wollen has not resigned his work without a parting warning as to the hospital's needs. It requires, he points out, an electric lift, a central heating system, and a new mortuary with a chapel. It also requires a debt of something like £300 to be paid off. Mr. Hugh Watkin, chairman of the board, has recently done his best to explain the hospital's outlook by making the annual meeting a reminder of their duty to the non-subscribing section—that is, we fear, the largest section of the residents. It is to be hoped that Mr. Husey may mark his first year of office by succeeding in realising some at least of the hospital's needs.

### A SANATORIUM FOR RAILWAYMEN.

Efforts are now being made by a committee of men from most of the principal railways to provide sanatorium benefits for railwaymen. According to Mr. George Bethel Bayley, "The idea is to make permanent provision for the free treatment of all cases in order of application—and without formality beyond a medical certificate. Sanatorium treatment is ordinarily restricted to early cases, but we propose also to make separate arrangements for those in the more advanced stage. There are over 90,000 members in the various railway superannuation funds, and if a good proportion come into the scheme this benefit can be provided on a sound actuarial basis." If the companies support the proposal a sanatorium for railway officials and clerks will probably be founded, but it is too early yet to describe the details of the scheme.

### THE KENSINGTON AND FULHAM GENERAL HOSPITAL.

As we go to press the management of the Kensington and Fulham General Hospital is submitting its annual report, which is satisfactory as showing not merely an increase in the number of patients, but a small balance in hand on the year's work. The feature on which the management most congratulates itself is the increase in the annual subscriptions, which is a very practical proof of the energy thrown into their work by Lord Claud Hamilton, the president; Lieutenant-Colonel H. J. F. Praeger, chairman of the board; and by the secretary, Mr. Louis M'Causland. The neighbourhood certainly seems to be rallying round this institution, which, having achieved one ambition by receiving a grant of £30 from the King's Fund, is anxious to fulfil another by reopening the children's ward. At present, we understand, the domestic staff has to sleep out. There seems, however, no doubt that the hospital is gaining the respect and regard of the boroughs which it serves, and if that is the case the fulfilment of its ambitions is only a matter of time.

### THE EFFECT OF AMALGAMATION AT BOURNEMOUTH.

The first report since the amalgamation of the two Bournemouth hospitals into the Royal Victoria and West Hants Hospital has peculiar interest. Dr. Roberts Thomson, chairman of the joint board of governors, stated at the annual meeting that the amalgamation gave cause for congratulation except from the present financial point of view, since there is, apart from legacies, a total deficit of nearly £3,000, the disheartening feature of which is the deficit of some £1,500 on the past year's working. It is interesting to hear that the working class " does a great deal," for Bournemouth is not regarded as a working-class centre. Hospital support, we imagine, should be an easy matter for such a well-off population. Mr. Godwin Pratt, chairman of the finance committee, produced some instructive figures, showing that while the amalgamation scheme had saved £64 in administration, £61 in surgical expenses, and £40 on establishment, apart from other matters, the income of the two hospitals had decreased by £425. This can only be due to two causes: to the conservatism of subscribers who have not grasped the necessity of continuing their subscriptions to the amalgamated hospital, or to a very grave indifference on the part of Bournemouth itself. No doubt Mr. Gordon Martyn has taken very great pains to see that no subscriber to either of the pre-existing institutions has escaped a reminder of his duty, and we can only hope that the name of the united hospital will soon triumph over any temporary lack of appeal which it may have to fight against at first.

### RAMSGATE HOSPITAL AND COMPLAINTS.

It is extraordinary on what similar lines complaints are brought forward against hospitals, and how the harmony of institutional management is impaired by indiscreet methods. The Ramsgate General Hospital is a case in point. Dr. C. H. Tamplin has recently drawn attention to certain alleged grievances, which he said had made the hospital unpopular in the town and which he thought ought to be looked into. Most people will agree with Dr. Tamplin's attitude, but a good deal of sympathy will also be felt with the hospital's president, Mr. Murray-Smith, who, while accepting the above principle, pointed out that the grievances should have been brought first to the notice of the committee before being urged at a public meeting. Mr. H. B. Hammond, the treasurer, also was obviously in the right in saying that complaints should be made at the time of the alleged grievance, and not postponed till six or eight months have passed. We mention these facts as showing all the usual want of care and lack of business sense which generally characterise complaints against hospitals. We hope, however, that these irritating defects in the presentation of the charges will not hinder their investigation, unnecessarily troublesome and difficult though that will now be. But it is surely strange that Dr. Tamplin, as a medical man and a hospital worker, should have adopted the tactics of the less well-informed layman in this matter.

### WOOD GREEN HOSPITAL DISPUTE.

A dispute has arisen between the medical staff of the Wood Green Cottage Hospital and the council, because the latter recently decided to increase the number of practitioners attending the hospital from twelve to eighteen. It has now been agreed, on the motion of Mr. F. S. Searle, the Southgate representative on the hospital council, that all the doctors of the district shall be entitled to attend their own patients after admission. Mr. Searle pointed out that when the hospital was opened the number of the staff was fixed at twelve, because there were only twelve doctors in the district. Now the population and the number of practitioners have increased, and those not on the staff were unwilling to send their patients to the hospital for fear of losing them, with the result that the hospital has been receiving practically the medical staff's patients alone. There seems little doubt that the new rule will solve the difficulty, which has been the almost inevitable outcome of the original rule. Such rules have been common cause of complaint, and the right remedy is to allow all doctors to attend their own patients here as elsewhere.

### A SUCCESSFUL COTTAGE HOSPITAL SECRETARY

Mr. H. F. Rogers, the recently appointed secretary of the Gorleston Cottage Hospital, Norfolk, has earned the warmest congratulations of everyone connected with the institution for the triumphant way in which, under his direction, the hospital for the first time in its history has shown a balance on the right side. His predecessors in the secretaryship never had the same success. Mr. J. Durrant, the hospital's chairman, said recently that " the new secretary has carried out his duties wonderfully well," so well indeed that the Rev. Forbes Phillips, the Vicar, has thought it wise to strike a warning note by remarking that hospital finance was like church finance in this—that it does not do to advertise too widely a balance on the right side. That no doubt is true, but if we mistake not, the supporters of this cottage hospital have been appealed to pretty frequently of recent years, and it is time for a new secretary to inaugurate his term of office by starting an endowment policy. This we are glad to see is to be acted upon by relegating a recent legacy to the hospital's invested funds.

### SWISS MEMORIAL TO AN ENGLISH DOCTOR.

The late Dr. Huggard, whose work as consulting practitioner and British Consul among the English colony at Davos Platz were described in these columns at the time of his death last October, is to have a memorial raised to him at Davos. An influential committee has been formed with two objects: "firstly, to place in the Queen Alexandra Sanatorium (an institution which largely owes its existence to his self-sacrificing philanthropy) a memorial tablet, if possible, with a medallion portrait of Dr. Huggard; and, secondly, to create a Huggard Benevolent Fund in aid of British invalids in Davos who are in need. . . . The fund to be invested in the names of trustees, and the income to be spent under the management of a carefully appointed local committee. It would be administered in close connection with the institution known as the English Nurses' Home, with which it may, if events prove it to be expedient, be afterwards incorporated and united." There is no doubt that it is on such lines as these that Dr. Huggard himself would have wished any such memorial to be undertaken; and it is equally certain that the number of poorer patients unable to afford to complete their cure is much larger than is realised, and should be, if possible, reduced. A Huggard Memorial on these lines will gain support wherever phthisical patients are found, so well is his name known in this migratory community of invalids. A strong local committee has taken the matter up, including such men as the Rev. E. S. Woods (English chaplain at Davos), Mr. B. Hudson (British Consul, Davos), Dr. J. Mitchell Bruce, Mr. Arnold F. Bill, and Mr. H. C. Wrinch. Subscriptions should be sent to Mr. Edward C. Simmons, 74 Cheapside, London, E.C., crossed "Huggard Memorial account."

### RESTRICTIONS ON THE SALE OF NARCOTICS.

The number of fatalities which have been recorded in recent years as a result of the consumption of soporific drugs is a full justification for the action now being taken by the Pharmaceutical Society. The Council of this body has recommended the Privy Council to add to the poisons schedule various drugs which are consumed in enormous quantities by the public. Sulphonal is already on the schedule, but many derivatives and allied products have not hitherto been included. Perhaps the best known of the drugs thus recommended for the schedule is veronal. This substance was introduced a few years ago, and was claimed to be quite innocuous; but the tale of its victims is an ever-lengthening one. As if to emphasise this, a fatal case of poisoning by veronal is recorded in last week's issue of the *Lancet*. It is high time that the public should realise the risk of self-drugging with these powerful hypnotics; and if the Privy Council accepts the recommendation of the Pharmaceutical Society, the consequent restrictions upon the sale of them will undoubtedly help to bring this about.

### THE FOWLER POSITION.

Nowadays hospital managers must keep well in touch with the new methods of treatment introduced by the medical staff. The evolution of aseptic surgery has entirely revolutionised the structure and equipment of the operating-theatre; ventilation and drainage are as important to the wards of a hospital as to a private house; advances in physiology have been the cause of new methods of invalid cookery needing special cooking utensils; and dressings are entirely different things from what they were twenty or even ten years ago. All these and many other factors have materially altered the construction and administration of the institution. We suppose that no improvement in medical and surgical treatment has had so great an influence in proportion to its simplicity as the introduction of the Fowler position. This is merely an upright or semi-upright position into which patients suffering from any acute inflammatory lesion of the abdomen are put, and all people on whom a laparotomy has been performed. The change in itself is simple, and apparently means very little. Its importance depends upon the diminished absorptive power of the pelvic peritoneum compared with that around the liver, and its increased bactericidal function. So important is this position that every patient in whom appendicitis is diagnosed should at once be put in it, although immediate operation be decided on, and performed in a few hours.

### THIS WEEK'S DRUG MARKET.

The depressing influence of the coal strike is beginning to be felt seriously in the drug and chemical markets; there has not been any noticeable interference with prices, but some delay in the transport of goods has been experienced, and it is said that some firms connected with the drug and chemical industries are already suffering from a shortage of coal-supply. As was anticipated, the price of glycerine has been reduced by ten shillings per cwt., and while a further reduction is not expected immediately it would not be altogether surprising if the refiners announce another fall in price at a not far distant date, in spite of the fact that the glycerin convention has not dissolved. Santonin is again dearer, and although it is reported that supplies are short, buyers would be well advised to buy only in very small quantities while extreme prices are ruling. Cod-liver oil is again lower in value, but it is thought that the lowest figure has not yet been touched; in another week or so it should be possible to judge approximately what the final price is likely to be, but in the meantime there should be no haste to buy. Opium continues to show a declining tendency, but the market is very quiet; morphine is also slightly lower in price, but no alterations in the quotations for codeine have been announced by the makers of that alkaloid. Menthol is rather dearer, as also is sarsaparilla. Cream of tartar is inclined to be slightly dearer.

